# Unconditional Cash Transfers and Prenatal Care Utilization in Flint, Michigan

**DOI:** 10.1001/jamanetworkopen.2025.38406

**Published:** 2025-10-20

**Authors:** Mona Hanna, Sumit Agarwal, H. Luke Shaefer

**Affiliations:** 1 Michigan State University–Hurley Children’s Hospital Pediatric Public Health Initiative, Charles Stewart Mott Department of Public Health, Michigan State University College of Human Medicine, Flint; 2 University of Michigan Medical School and School of Public Health, Ann Arbor; 3 Gerald R. Ford School of Public Policy and Poverty Solutions, University of Michigan, Ann Arbor

## Abstract

This cohort study examines prenatal care utilization before and after implementation of an unconditional cash transfer program in Flint, Michigan, compared with similar cities in Michigan.

## Introduction

Timely prenatal care is important for improving maternal and infant health.^1,2^ As expenses increase and earnings decrease, the perinatal period of pregnancy and infancy is marked by acute economic insecurity, which underlies suboptimal prenatal care utilization.^3^ Few interventions proactively address economic hardship during this period.

In 2024, an unconditional cash transfer program, Rx Kids, launched in Flint, Michigan, providing every expectant mother—upon verification of pregnancy and gestational age by health care practitioners—$1500 midpregnancy and $500 monthly after birth until age 1 year. It is the first community-wide cash transfer program in the US with universal eligibility (ie, no means testing) targeting the perinatal period. We examined prenatal care utilization in Flint before and after implementation of Rx Kids compared with similar cities in Michigan.

## Methods

This cohort study adheres to STROBE reporting guidelines and was approved by the institutional review board at Michigan State University with a waiver of informed consent. To evaluate Rx Kids (eAppendix 1 in Supplement 1), we analyzed statewide vital records data derived from birth certificates. We identified all births from January 2021 through March 2025 in Flint and 21 matched cities and, for each half-year, estimated rates of prenatal care adequacy according to the Kessner index and its components (eAppendix 2 in Supplement 1). The Kessner index incorporates the timing of prenatal care initiation, visit quantity, and gestational age at birth to classify care as adequate or inadequate. A difference-in-differences strategy was used to estimate the adjusted mean change in outcomes in Flint relative to the matched cities, before and after program implementation in January 2024 (eAppendix 3 in Supplement 1). Linear regression models were fitted with an interaction term between indicators for Flint residency and after implementation, adjusting for birth month, mother’s age, education, marital status, and race and ethnicity, in addition to city and year fixed effects.

## Results

Our study population included 32 157 births, including 4264 in Flint. The mean (SD) maternal age was 27.7 (5.8) years, 3761 mothers (11.7%) were Hispanic, 14 299 (44.5%) were non-Hispanic Black, 12 076 (37.6%) were non-Hispanic White, 2021 (6.3%) were other races (American Indian or Alaskan Native, Asian or Pacific Islander, multiracial, and other races not otherwise listed), and 21 053 (65.8%) were Medicaid insured (Table). Comparing Rx Kids enrollment numbers with birth certificate data showed an aggregate take-up rate of approximately 100%, with more than 90% enrolling prenatally. There was no evidence of differing trends in prenatal care adequacy before 2024 between Flint and the matched cities (β = −0.00545; 95% CI, −0.055 to 0.044; *P* = .82).

**Table.** Characteristics of the Study Population

Characteristic	Participants, No. (%)		
	Flint (n = 4264)	Comparison cities (n = 27 893)	Overall (N = 32 157)
Maternal age, y			
16-19	349 (8.2)	1853 (6.6)	2202 (6.9)
20-24	1219 (28.6)	6840 (24.5)	8059 (25.1)
25-29	1277 (30.0)	8384 (30.1)	9661 (30.1)
30-34	962 (22.6)	7071 (25.4)	8033 (25.0)
35-39	374 (8.8)	3045 (10.9)	3419 (10.6)
≥40	82 (1.9)	694 (2.5)	776 (2.4)
Total with data, No.	4263	27 887	32 150
Maternal race and ethnicity^a^			
Hispanic	219 (5.1)	3542 (12.7)	3761 (11.7)
Non-Hispanic Black	2488 (58.4)	11 811 (42.3)	14 299 (44.5)
Non-Hispanic White	1378 (32.3)	10 698 (38.4)	12 076 (37.6)
Other	179 (4.2)	1842 (6.6)	2021 (6.3)
Total with data, No.	4264	27 893	32 157
Maternal education			
Less than high school	1018 (24.0)	4713 (17.1)	5731 (18.0)
High school or equivalent	1443 (34.0)	10 421 (37.9)	11 864 (37.4)
Some college	1269 (29.9)	6391 (23.2)	7660 (24.1)
Associate degree	239 (5.6)	1721 (6.3)	1960 (6.2)
College degree or more	277 (6.5)	4269 (15.5)	4546 (14.3)
Total with data, No.	4246	27 515	31 761
Marital status			
Married	727 (17.1)	9245 (33.2)	9972 (31.0)
Not married	3535 (82.9)	18 631 (66.8)	22 166 (69.0)
Total with data, No.	4262	27 876	32 138
Primary payer			
Medicaid	3594 (84.6)	17 459 (62.9)	21 053 (65.8)
Commercial insurance	621 (14.6)	9444 (34.0)	10 065 (31.5)
Other	34 (0.8)	849 (3.1)	883 (2.8)
Total with data, No.	4249	27 752	32 001
Parity (prior births)			
0	1351 (31.8)	9882 (35.5)	11 233 (35.0)
1	1078 (25.4)	7705 (27.7)	8783 (27.4)
2	800 (18.8)	4804 (17.3)	5604 (17.5)
3	506 (11.9)	2766 (10.0)	3272 (10.2)
≥4	516 (12.1)	2649 (9.5)	3165 (9.9)
Total with data, No.	4251	27 806	32 057

^a^ Maternal race and ethnicity, which may capture social factors and experience of structural racism, are reported as documented in the vital records derived from birth certificates. The other category includes American Indian or Alaskan Native, Asian or Pacific Islander, multiracial, and other races not otherwise listed.

Before Rx Kids, the proportion of births in Flint with adequate prenatal care was 50.5% (1490 of 2952 births). After program launch, the gap narrowed, and in 2025, the proportion of births in Flint with adequate prenatal care surpassed the matched cities (Figure). This pattern corresponded to an adjusted increase of 9.1 percentage points (95% CI, 5.6 to 12.5 percentage points) in Flint after Rx Kids, compared with matched cities. The baseline proportion of births in Flint with no prenatal care was 4.5% (133 of 2934 births), the mean (SD) number of visits was 11.2 (5.4), and 59.2% of births (1655 of 2795 births) had initiation of prenatal care in the first trimester. Compared with matched cities, implementation of Rx Kids in Flint was associated with improvements in the proportion of births with no prenatal care (−1.9 percentage points; 95% CI, −2.6 to −1.2 percentage points), number of visits (0.8 visits; 95% CI, 0.2 to 1.4 visits), and initiation of prenatal care in the first trimester (5.6 percentage points; 95% CI, 2.5 to 8.7 percentage points).

**Figure.**
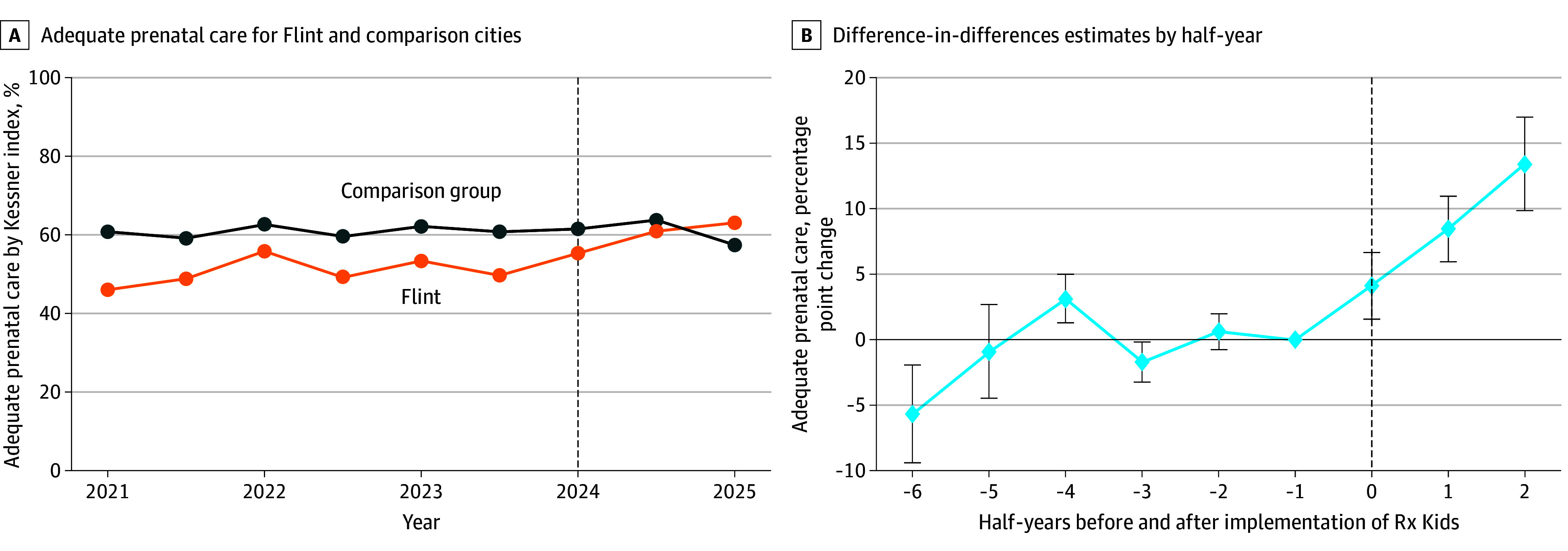
Prenatal Care Adequacy Before and After Implementation of the Rx Kids Cash Transfer Program Graphs show data for Flint vs comparison group of matched cities (A) and difference-in-differences estimates by half-year (B) before and after implementation of Rx Kids in January 2024. Dashed vertical lines in both panels denote the date of implementation. Error bars in panel B denote 95% CIs.

## Discussion

This cohort study found that implementation of Rx Kids, an upstream and place-based treatment of perinatal economic hardship, was associated with substantial improvements in prenatal care utilization in Flint, Michigan. The monetary support, including its anticipation, may have buffered the economic shock of pregnancy to help cover the direct or indirect costs associated with accessing care.^4^

Limitations include generalizability and lack of granular information on the content or quality of prenatal care. Strengths include the high take-up rate of the program and use of rigorous quasiexperimental methods. These results underscore the importance of economic security in the perinatal period, which could have broad implications for improving maternal-infant health.^5,6^

## Notes

Supplement 1. eAppendix 1. Additional details about the Rx Kids intervention eAppendix 2. Additional details about the data source and outcomes eAppendix 3. Additional details about the analytic specification eReferences

Supplement 2. Data Sharing Statement
