# Supplementary material for: Unconditional Cash Transfers and Prenatal Care Utilization in Flint, Michigan
Source: JAMA Netw Open. 2025 Oct 20;8(10):e2538406. doi: 10.1001/jamanetworkopen.2025.38406 (PMC12538360; doi:10.1001/jamanetworkopen.2025.38406)
Supplement: Supplement 1. — eAppendix 1. Additional details about the Rx Kids intervention eAppendix 2. Additional details about the data source and outcomes eAppendix 3. Additional details about the analytic specification eReferences [file jamanetwopen-e2538406-s001.pdf]

Supplemental Online Content

Hanna M, Agarwal S, Shaefer HL. Unconditional cash transfers and prenatal care utilization in Flint, Michigan. *JAMA Netw Open*. 2025;8(10):e2538406. doi:10.1001/jamanetworkopen.2025.38406

eAppendix 1. Additional details about the Rx Kids intervention

eAppendix 2. Additional details about the data source and outcomes

eAppendix 3. Additional details about the analytic specification

eReferences

This supplemental material has been provided by the authors to give readers additional information about their work.

© 2025 Hanna M et al. *JAMA Network Open*.

eAppendix 1: Additional details about the Rx Kids intervention

In the city of Flint, Michigan, the poverty rate for 2023 was 34%, about three times that of the national average, with even higher rates for child poverty. Routinely among the poorest cities in the nation, Flint became recognized nationally in 2015 after attention was brought to contamination of its drinking water.[1-3] The high rates of poverty contribute to adverse maternal-infant health outcomes and racial inequities in perinatal health. To address how poverty and the economic shock of pregnancy influence child development starting in utero and the first year of life,[4-8] the "Rx Kids" cash prescription program was launched and began enrolling participants in January 2024. It is the first community-wide cash transfer program in the country with universal eligibility (i.e., no means testing) that targets the perinatal period before and after birth. Recruitment materials were distributed at multiple community touchpoints, including prenatal clinics, as well as WIC offices, religious institutions, nonprofits, and more. Mothers most frequently reported hearing about the program from friends, family, or neighbors, social media, and traditional outreach methods, including prenatal clinics, program workers, and recruitment materials at other places. Eligible participants enroll online and must provide documentation of their age, residency, and pregnancy status as confirmed by a medical provider. After enrollment, the program provides participants with $1,500 during pregnancy (i.e., after 20 weeks' gestation in 2024 and after 16 weeks' gestation in 2025) and $500 per month for twelve months after birth, totaling $7,500 in unconditional cash and made available via direct deposit to a bank account or prepaid debit card. As a non-taxable gift to beneficiaries, the cash transfer is not subject to income taxes and does not affect eligibility for most other public benefits, including Medicaid and the Supplemental Nutrition Assistance Program.[9]

eAppendix 2: Additional details about the data source and outcomes

The administrative birth records data used in this study were obtained from the Division for Vital Records and Health Statistics in the Michigan Department of Health and Human Services. Our study population consisted of all births from January 2021 through March 2025 among women aged 16 years and older in the city of Flint, Michigan, and other cities in Michigan that were similar to Flint based on population size, poverty rate, and racial composition. Births were geocoded based on the mother's residential address. Matched cities included those with a population of 5,000 to 125,000, a poverty rate of greater or equal to 15%, and a non-Hispanic Black population of at least 20%, as determined using the U.S. Census Bureau's 2019-2023 American Community Survey.[10] After excluding areas adjacent to the city of Flint such as Beecher and Flint Township as well as Kalamazoo which had its own perinatal programming during our study period, the twenty-one cities that met the match criteria included Albion, Benton Harbor, Benton Township, Bridgeport Township, Buena Vista Township, Eastpointe, Ecorse, Harper Woods, Highland Park, Inkster, Jackson, Lansing, Muskegon, Muskegon Heights, Pontiac, River Rouge, Saginaw, St. Louis (in Michigan), Wayne, Ypsilanti, and Ypsilanti Township. These comparison cities are somewhat more advantaged than Flint as measured by educational attainment and insurance status (see Table in main text). They represent the closest comparisons available in the state.

Our primary outcome was a binary measure for whether a birth had adequate prenatal care as classified by the Kessner index. The Kessner index is a commonly used classification system for determining prenatal care adequacy that incorporates both the timing of the initial prenatal care visit and quantity of visits for a given gestational age at birth.[11] For example, a term birth for which prenatal care was initiated in the first trimester and had at least nine prenatal

© 2025 Hanna M et al. *JAMA Network Open*.

visits would be classified as adequate. There are well-known limitations of this classification system,[12] but we specifically chose the Kessner index because of its relative simplicity, greater stability in analyses like ours relying on serial cross-sectional data, and heavier emphasis on first trimester initiation. Furthermore, new 2025 guidelines from the American College of Obstetricians and Gynecologists recommend six to ten visits for average-risk individuals,[13] which is consistent with the range of visits considered under the Kessner index and fewer than the former recommendation of 12-14 visits upon which the alternative Adequacy of Prenatal Care Utilization Index (APNCU index, also known as the Kotelchuck index) is traditionally based. Missingness was minimal (less than 0.01%). Although there may be measurement error in the vital records, data quality improved substantially after the 2003 revision of the U.S. Standard Certificate of Live Birth. The prenatal care measures have been validated against medical records,[14,15] and our estimates would remain unbiased under the standard assumptions of our difference-in-differences methodology described below. To aid interpretation of prenatal care adequacy, we separately examined the underlying components of the Kessner index, including a binary measure for whether there was no prenatal care, a count measure for the quantity of visits over the prenatal period, and a binary measure for prenatal care initiation in the first trimester.

eAppendix 3. Additional details about the analytic specification

We first plotted the unadjusted proportion of births for which there was adequate prenatal care in each half-year starting in 2021 for Flint, Michigan, and separately for the comparison group of matched cities. Our data comprised births through March 2025, so the unadjusted proportion included the first three months of the year for 2025. We then used a difference-in-differences strategy to compare outcomes in Flint where Rx Kids was implemented starting in

© 2025 Hanna M et al. *JAMA Network Open*.

January 2024 to that of the comparison cities. Specifically, we fit the following linear regression model for each outcome: $y_{ict} = \beta_0 + \beta_1(FLINT_c * POST_t) + \boldsymbol{X'}_{ict}\beta + \delta_c + \gamma_t + \varepsilon_{ict}$, where y_{ict} denotes the outcome for individual i in city c and year t, X'_{ict} is a vector of covariates including birth month as well as maternal characteristics in categories (i.e., age, education, marital status, and race/ethnicity), δ_c are city fixed effects, and γ_t are year fixed effects. The coefficient of interest is β_1 on the interaction term between living in Flint after implementation of Rx Kids. Because Rx Kids is a citywide intervention, standard errors were clustered at the city level.

The difference-in-differences strategy is unbiased with respect to unmeasured confounders that are time-invariant as well as those that are time-varying with consistent trends between groups. The key causal assumption underlying our difference-in-differences strategy was that the outcomes for Flint would have evolved similarly (i.e., would have been parallel) to those of the comparison group of cities had it not been for the Rx Kids program. We assessed the plausibility of this "parallel trends" assumption visually and by explicitly testing for differential trends between the two groups prior to implementation of Rx Kids, which failed to reject the null hypothesis of parallel trends during the pre-period (i.e., prior to 2024) between Flint and the comparison group of cities in each of the outcomes (P=0.82 for prenatal adequacy, P=0.17 for no prenatal care, P=0.58 for number of visits, and P=0.98 for initiation in the first trimester). We also examined an event-study specification of the difference-in-difference analysis in which the Flint indicator in the equation above is interacted with each year before and after implementation of Rx Kids. We also queried leaders across multiple sectors, and no other major changes were identified in economic conditions or prenatal supports and services that would coincide with the timing of the implementation of Rx Kids in Flint.

© 2025 Hanna M et al. *JAMA Network Open*.

In a comparison of enrollment numbers in Rx Kids to birth counts at the aggregate and monthly levels, the take-up rate was estimated to be complete at 100%, with more than 90% enrolling prenatally. To account for the ramp-up period in prenatal enrollment, we applied weights of less than one with a monotonic increase for observations from January through May 2024. Finally, we performed several sensitivity analyses to test the robustness of our results. First, we ensured that our results were robust to alternative model specifications, including a logistic model with computed predicted probabilities as well as models that excluded any weights and covariates. Second, to account for any potential spillovers or threats to the stable unit treatment value assumption, we assessed for compositional changes in who gives birth and repeated our analyses to include surrounding communities of Flint as part of the intervention group. There was no evidence of compositional change based on maternal demographics, including age, education, marital status, and race/ethnicity; the results of the other sensitivity analyses were consistent with the main results. All analyses were performed in Stata version 18.0, and the 95% confidence intervals are reflective of 0.025 in each tail.

eReferences

1. Hanna-Attisha M, LaChance J, Sadler RC, Champney Schnepp A. Elevated Blood Lead Levels in Children Associated With the Flint Drinking Water Crisis: A Spatial Analysis of Risk and Public Health Response. *Am J Public Health*. 2016;106(2):283-290. doi:10.2105/AJPH.2015.303003
2. Hanna M. *What the Eyes Don't See: A Story of Crisis, Resistance, and Hope in an American City*. One World; 2018.

© 2025 Hanna M et al. *JAMA Network Open*.

3. Bellinger DC. Lead Contamination in Flint — An Abject Failure to Protect Public Health. *N Engl J Med*. 2016;374(12):1101-1103. doi:10.1056/NEJMp1601013
4. Stanczyk AB. The Dynamics of U.S. Household Economic Circumstances Around a Birth. *Demography*. 2020;57(4):1271-1296. doi:10.1007/s13524-020-00897-1
5. McConnell M, Agarwal S, Hanson E, McCrady E, Parker MG, Bona K. Prescription for Cash? Cash Support to Low-Income Families in Maternal and Pediatric Health Care Settings. *Milbank Quarterly*. 2024;102(1):64-82. doi:10.1111/1468-0009.12679
6. Hamilton C, Sariscsany L, Waldfogel J, Wimer C. Experiences of Poverty Around the Time of a Birth: A Research Note. *Demography.* 2023;60(4):965-976. doi:10.1215/00703370-10837403
7. Marti-Castaner M, Pavlenko T, Engel R, et al. Poverty after Birth: How Mothers Experience and Navigate U.S. Safety Net Programs to Address Family Needs. *J Child Fam Stud.* 2022;31(8):2248-2265. doi:10.1007/s10826-022-02322-0
8. Shaefer HL, Hanna M, Harris D, Richardson D, Laker M. Protecting the health of children with universal child cash benefits. *The Lancet*. 2024;404(10469):2380-2391. doi:10.1016/S0140-6736(24)02366-3
9. Hanna M, Shaefer HL. *Playbook for Replicating Rx Kids: Utilizing TANF and Protecting Public Benefits.* 2024. rxkids.org/impact/playbook/
10. U.S. Census Bureau. American Community Survey 5-Year Data (2009-2023). Published online December 12, 2024. Accessed January 5, 2025. https://www.census.gov/data/developers/data-sets/acs-5year.html

© 2025 Hanna M et al. *JAMA Network Open*.

11. National Research Council. *Infant Death: An Analysis by Maternal Risk and Health Care: Contrasts in Health Status Volume 1*. (Kessner D, ed.). The National Academies Press; 1973. doi:10.17226/28722
12. Kogan MD, Martin JA, Alexander GR, Kotelchuck M, Ventura SJ, Frigoletto FD. The Changing Pattern of Prenatal Care Utilization in the United States, 1981-1995, Using Different Prenatal Care Indices. JAMA. 1998;279(20):1623–1628.
13. Committee on Clinical Consensus-Obstetrics. *Tailored Prenatal Care Delivery for Pregnant Individuals.* ACOG Clinical Consensus. 2025;145(5). https://www.acog.org/clinical/clinical-guidance/clinical-consensus/articles/2025/04/tailored-prenatal-care-delivery-for-pregnant-individuals
14. Gregory ECW, Martin JA, Argov EL, Osterman MJK. Assessing the Quality of Medical and Health Data From the 2003 Birth Certificate Revision: Results From New York City. *Natl Vital Stat Rep.* 2019;68(8):1-20.
15. Martin JA, Wilson EC, Osterman MJK, Saadi EW, Sutton SR, Hamilton BE. Assessing the quality of medical and health data from the 2003 birth certificate revision: results from two states. *Natl Vital Stat Rep*. 2013;62(2):1-19

© 2025 Hanna M et al. *JAMA Network Open*.
