# Supplementary material for: Unconditional Cash Transfers and Prenatal Care Utilization in Flint, Michigan
Source: JAMA Netw Open. 2025 Oct 20;8(10):e2538406. doi: 10.1001/jamanetworkopen.2025.38406 (PMC12538360; doi:10.1001/jamanetworkopen.2025.38406)
Supplement: Supplement 2. — Data Sharing Statement [file jamanetwopen-e2538406-s002.pdf]

Data Sharing Statement

Hanna. Unconditional Cash Transfers and Prenatal Care Utilization in Flint, Michigan. *JAMA Netw Open*. Published October 20, 2025. doi:10.1001/jamanetworkopen.2025.38406

Data
Data available: No

Additional Information
Explanation for why data not available: The data is not available. The data for this study came from the Michigan Department of Health and Human Services.
